# Serum Vitamin D Levels Were Not Associated With the Risk of Aneurysmal Subarachnoid Hemorrhage: A Large Cohort Study With Propensity Score Matching and Mendelian Randomization Analysis

**DOI:** 10.1111/cns.70617

**Published:** 2025-09-30

**Authors:** Haoran Qiu, Kai Chen, Yang Yu, Ziyin Song, Jingzheng Liu, Lvyin Luo, Xinlong Ma, Zhaoyang Yuan, Maogui Li, Jianfeng Zhuang, Mingxiang Zhang, Wandong Su, Yunyan Wang, Donghai Wang, Weiying Zhong

**Affiliations:** ^1^ Department of Neurosurgery, Qilu Hospital, Cheeloo College of Medicine and Institute of Brain and Brain‐Inspired Science Shandong University Jinan China; ^2^ Cheeloo College of Medicine Shandong University Jinan China; ^3^ Key Laboratory of Cardiovascular Remodeling and Function Research Chinese Ministry of Education, Chinese Ministry of Health, and Chinese Academy of Medical Sciences, Qilu Hospital of Shandong University Jinan China

**Keywords:** intracranial aneurysm, Mendelian randomization, propensity score matching, subarachnoid hemorrhage, vitamin D

## Abstract

**Background:**

Vitamin D (VitD) may protect arterial health, but its link to cerebral aneurysm rupture remains unclear. This study aims to investigate the correlation between serum VitD levels and the risk of aneurysmal subarachnoid hemorrhage (aSAH).

**Methods:**

This retrospective study included patients with ruptured or unruptured aneurysms treated between 2018 and 2021. Univariate and multivariate analyses were conducted. Propensity score matching (PSM) analysis was conducted to balance confounding factors. Furthermore, the causal relationship between VitD and aSAH was investigated using a two‐sample Mendelian randomization (MR) analysis. Summary‐level data for the exposure (VitD) and outcome (aSAH) were obtained from public genome‐wide association study datasets. Multiple MR methods, including inverse variance weighted, MR‐Egger, weighted median, simple mode, and weighted mode, were utilized to assess causality. Sensitivity analyses were conducted to evaluate the robustness and reliability of the causal estimates.

**Results:**

A total of 499 patients with 619 intracranial aneurysms were included. Among them, 337 (68%) were female, 184 cases (36.9%) had ruptured aneurysms, and 105 (21.0%) had multiple aneurysms. VitD levels and VitD deficiency showed no association with aSAH in univariate or multivariate analysis before PSM (*p* > 0.05). After PSM (112 matched aSAH patients), VitD levels and VitD deficiency remained unassociated with the risk of aSAH (*p* = 0.947). MR analysis, including inverse variance weighted methods, found no causal link between VitD levels and aSAH (OR: 1.00; 95% CI: 0.82–1.23; *p* = 0.966).

**Conclusion:**

This study found that serum VitD levels are neither associated with nor causally linked to aSAH. The inverse associations observed in previous studies may be attributed to confounding factors or reverse causation. A prospective, large‐scale study with long‐term follow‐up is warranted to validate these findings.

## Graphical abstract

No significant association or causal relationship was observed between serum vitamin D levels and aneurysmal subarachnoid hemorrhage (aSAH).

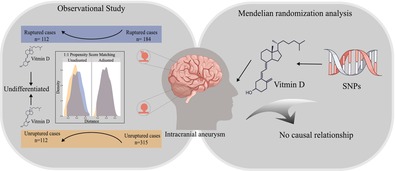

## 1 Introduction

Intracranial aneurysms (IAs) are abnormal dilations of the cerebral arterial wall, posing a significant health risk due to their potential rupture, which can cause subarachnoid hemorrhage (SAH), a life‐threatening condition with high morbidity and mortality [1]. Incidental IAs are increasingly diagnosed owing to the widespread use of noninvasive neuroimaging. However, not all unruptured IAs rupture during a patient's lifetime. Clinical factors such as hypertension, smoking, history of SAH, as well as aneurysm size and location, have been associated with IA growth and rupture [2, 3]. Tools such as the ELAPSS and PHASES scores, which incorporate these factors, have been developed to predict the risk of IA growth and rupture [4, 5]. Nevertheless, the literature lacks appropriate hematological indices for predicting aneurysm rupture.

While the pathological mechanisms underlying IA rupture remain incompletely understood, factors such as inflammation, oxidative stress, hemodynamic stress, and genetic predispositions are considered contributing factors [2, 3]. Abnormal blood flow and shear stress may compromise vascular integrity, while inflammation and oxidative stress could further damage the aneurysm wall, thereby increasing the rupture risk [2, 3, 6]. Vitamin D (VitD), a fat‐soluble steroid vitamin essential for calcium homeostasis and immune regulation, may confer vascular health benefits due to its anti‐inflammatory and antioxidative properties [7, 8]. Low levels of VitD are associated with elevated vascular inflammation, oxidative stress, endothelial dysfunction, and increased risk of cardiovascular and cerebrovascular events, including hypertension, atherosclerosis, myocardial infarction, and stroke [7, 8, 9]. VitD deficiency is also prevalent among IA patients [10, 11]. However, its relationship with IA rupture remains unclear. One study reported an inverse correlation between VitD levels and the risk of aneurysmal subarachnoid hemorrhage (aSAH) [12], whereas another found no association [11]. These discrepancies may arise from variations in sample size, study populations, methodologies, or unaccounted confounders. Additionally, VitD deficiency could result from aneurysm rupture, suggesting potential reverse causality. Thus, the specific role of VitD in aSAH remains controversial, and further research is required to determine whether VitD deficiency is associated with IA rupture.

Therefore, in this study, a large case–control study with propensity score matching (PSM) to balance confounding factors was conducted to investigate the potential association between VitD levels and aSAH. However, observational studies remain vulnerable to unmeasured confounders. To address this limitation, we also performed a two‐sample Mendelian randomization (MR) analysis to explore potential causal relationships. By integrating these methods, this study aims to provide high‐quality evidence regarding the relationship between VitD status and aSAH. The findings may improve our understanding of IA rupture mechanisms.

## 2 Materials and Methods

### 2.1 Observational Study Design

**FIGURE 1:**
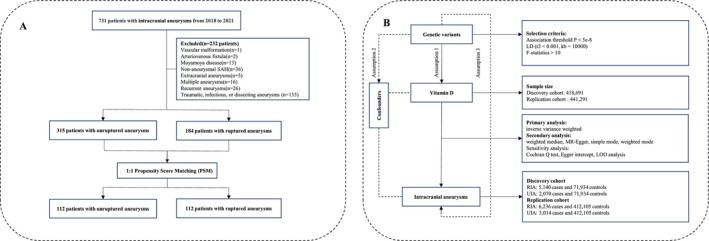
The flow chart of the present study.

This retrospective case–control study comprised consecutive patients with IA treated at Qilu Hospital of Shandong University between March 2018 and May 2021. The study was approved by the Ethics Committee of Qilu Hospital of Shandong University, and written informed consent was obtained from all participants prior to enrollment. All IAs were confirmed using computed tomography (CT), magnetic resonance imaging (MRI), or digital subtraction angiography (DSA). SAH was confirmed via CT scans. Ruptured IAs were defined as cases presenting with aneurysmal SAH, regardless of the number of aneurysms present, while unruptured IAs were defined as patients with at least one aneurysm and no history of SAH. Exclusion criteria were as follows: (1) recent administration of VitD supplements or glucocorticoids within 1 month prior to the study; (2) concomitant vascular diseases, such as arteriovenous malformations, moyamoya disease, or arteriovenous fistulas; (3) recurrent, fusiform, traumatic, bacterial, or dissecting aneurysms; (4) aneurysmal SAH occurring more than 72 h before admission; (5) SAH of unknown etiology or non‐aneurysmal SAH; and (6) age < 18 years. Ultimately, 499 patients with either unruptured or ruptured IAs were included in the study. The study flowchart is presented in Figure 1A.

The patients' demographic characteristics, comorbidities (including diabetes mellitus, hypertension, and hyperlipidemia), and aneurysm characteristics (such as location and number) were recorded. For patients with multiple aneurysms, the study evaluated the location (anterior or posterior circulation) of the primary aneurysm treated during hospitalization. Fasting venous blood samples were collected within 24 h of admission to measure 25‐hydroxyvitamin D levels and conduct lipid profile tests, including triglycerides (TG), total cholesterol (TC), low‐density lipoprotein cholesterol (LDL‐C), and high‐density lipoprotein cholesterol (HDL‐C). Body mass index (BMI) was categorized as underweight (≤ 18.5 kg/m^2^), normal weight (18.5–24 kg/m^2^), overweight (24–28 kg/m^2^), or obese (> 28 kg/m^2^). Hypertension was defined as a documented history of the condition (systolic blood pressure ≥ 140 mmHg or diastolic blood pressure ≥ 90 mmHg), irrespective of antihypertensive treatment. Diabetes mellitus was defined as patients receiving antidiabetic medication, a fasting glucose level ≥ 7 mmol/L from previous health assessments, or a hemoglobin A1c level ≥ 6.5%. Hyperlipidemia was defined as lipid‐lowering medication use or fasting plasma TC ≥ 6 mmol/L, TG ≥ 2 mmol/L, or LDL ≥ 3.5 mmol/L. Current smoking was defined as consumption of ≥ 5 cigarettes per day, and alcohol consumption as intake of ≥ 150 g per week. VitD levels were categorized as deficient (< 20 ng/mL), insufficient (20–29 ng/mL), or normal (≥ 30 ng/mL).

Statistical analyses were performed using SPSS 25.0 (IBM Corp.). Continuous variables were expressed as mean ± standard deviation or median [interquartile range], depending on their distribution. The Shapiro–Wilk test was used to assess normality, with a *p*‐value < 0.05 indicating a deviation from normal distribution. Group comparisons were conducted using Student's *t*‐test for normally distributed variables and the Mann–Whitney *U* test for non‐normally distributed variables. Categorical variables were expressed as frequencies and percentages and were compared using the *χ* ^2^ test or Fisher's exact test. Factors with *p* < 0.1 in univariate analysis were included in binary logistic regression analysis using an enter process to identify independent predictors. PSM was used to balance confounding factors between patients with and without aneurysmal SAH. A one‐to‐one PSM analysis using the nearest neighbor method with a caliper value of 0.01 was conducted to adjust for imbalanced factors. A restricted cubic spline (RCS) regression model was employed to examine potential linear or nonlinear associations between serum VitD levels and the risk of aSAH. Four knots were positioned at the 5th, 35th, 65th, and 95th percentiles of the VitD distribution, with the median serving as the reference point. The RCS model was fitted using the “rms” package (version 6.8–2), and spline plots were generated using the “plotRCS” package (version 0.1.5) in R software (version 4.4.1). Statistical significance was defined as a two‐tailed *p*‐value < 0.05.

### 2.2 MR Analysis

A two‐sample MR analysis was performed to investigate the causal relationship between VitD levels and aSAH. Figure 1B shows the analysis flowchart.

Genetic instruments for VitD levels were obtained from genome‐wide association studies (GWAS) datasets available through the IEU OpenGWAS project (https://gwas.mrcieu.ac.uk/), a public resource for investigating genetic associations with complex traits. Summary‐level GWAS data on IAs were derived from two large cohorts: the discovery cohort, conducted by the International Stroke Genetics Consortium (ISGC) with individuals of European ancestry, and the replication cohort from FinnGen. The discovery cohort included 5140 ruptured cases, 2070 unruptured cases, and 71,934 controls, while the replication cohort had 6236 ruptured cases, 3014 unruptured cases, and 412,105 controls. Additional details regarding the data sources used in this study are provided in File S1.

From these GWAS datasets, single nucleotide polymorphisms (SNPs) significantly associated with VitD levels (*p* < 5 × 10^−8^) were selected as candidate instrumental variables (IVs). Linkage disequilibrium (LD) analysis was conducted to ensure SNP independence, applying a threshold of LD‐*r* ^2^ < 0.001 and a clumping distance threshold of > 10,000 kb. The *F*‐statistic [*F* = *R* ^2^(N‐2)/(1‐*R* ^2^)] was calculated to evaluate IV strength, with SNPs failing to meet the criterion of *F* ≥ 10 excluded to mitigate weak instrument bias, thereby maintaining the robustness of the analysis.

The inverse variance‐weighted (IVW) method was employed as the primary approach for causal estimation, providing reliable results in the absence of heterogeneity or horizontal pleiotropy. Supplementary methods—including MR‐Egger regression, weighted median, simple mode, and weighted mode—were used to ensure robustness under varying conditions. Heterogeneity was assessed using Cochran's *Q* test, and if heterogeneity was detected (*p* < 0.05), adjusted results were reanalyzed [13]. Horizontal pleiotropy was evaluated using the MR‐Egger intercept method, with *p* > 0.05 indicating no pleiotropy and supporting MR reliability [14]. The leave‐one‐out (LOO) method was applied to systematically exclude each SNP to assess result stability. All analyses were conducted using R software (version 4.4.1) with the “TwoSampleMR” package [15], with statistical significance defined as *p* < 0.05.

## 3 Results

### 3.1 Associations of Vitamin D With IA Rupture in the Observational Study

The baseline characteristics are presented in Table 1. A total of 499 patients with 619 IAs were included in this study, comprising 337 (67.5%) females and 162 (32.5%) males. The median age was 58.0 years [52.0, 64.0], and the median BMI was 24.8 kg/m^2^ [22.3, 27.3]. Among these patients, 184 (36.9%) had ruptured IAs, and 105 (21.0%) had multiple IAs.

**TABLE 1:** Baseline information of enrolled patients before or after propensity score matching (PSM).

Characteristic	Before PSM				After PSM			
	Overall (*n* = 499)	Unruptured (*n* = 315)	Ruptured (*n* = 184)	*p*	Overall (*n* = 224)	Unruptured (*n* = 112)	Ruptured (*n* = 112)	*p*
Sex (Female)	337 (67.5)	215 (43.1)	122 (24.4)	0.654^a^	157 (70.1)	80 (35.7)	77 (34.4)	0.662^a^
Age (year)				0.026^a^				0.774^a^
≤ 50	110 (22.0)	58 (11.6)	52 (10.4)		43 (19.2)	24 (10.7)	19 (8.5)	
51–60	178 (35.7)	110 (22.0)	68 (13.6)		85 (37.9)	41 (18.3)	44 (19.6)	
61–70	168 (33.7)	118 (23.6)	50 (10.0)		78 (34.8)	37 (16.5)	41 (18.3)	
> 70	43 (8.6)	29 (5.8)	14 (2.8)		18 (8.0)	9 (7.3)	10 (8.1)	
BMI				0.699^a^				0.625^b^
Underweight	15 (3.0)	9 (1.8)	6 (1.2)		7 (3.1)	3 (1.3)	4 (1.8)	
Normal	188 (37.9)	114 (22.8)	75 (15.0)		89 (39.7)	48 (21.4)	41 (18.3)	
Overweight	204 (40.7)	134 (26.9)	69 (13.8)		94 (42.0)	47 (21.0)	47 (21.0)	
Obese	92 (18.4)	58 (11.6)	34 (6.8)		34 (15.2)	14 (6.3)	20 (8.9)	
Smoking	85 (17.0)	50 (10.0)	35 (7.0)	0.367^a^	36 (16.1)	19 (8.5)	17 (7.6)	0.716^a^
Drinking	53 (10.6)	29 (5.8)	24 (4.8)	0.180^a^	25 (11.2)	11 (4.9)	14 (6.3)	0.524^a^
Diabetes	70 (14.0)	53 (10.6)	17 (3.4)	0.019^a^	20 (8.9)	9 (4.0)	11 (4.9)	0.639^a^
Hypertension	291 (58.3)	184 (36.9)	107 (21.4)	0.955^a^	132 (58.9)	63 (28.1)	69 (30.8)	0.415^a^
Hyperlipidemia	142 (28.5)	91 (18.2)	51 (10.2)	0.780^a^	56 (25.0)	28 (12.5)	28 (12.5)	1.000^a^
Blood test dates				0.147^a^				0.885^a^
Mar–May	130 (26.1)	81 (16.2)	49 (9.8)		60 (26.8)	28 (12.5)	32 (14.3)	
Jun–Aug	100 (20.0)	57 (11.4)	43 (8.6)		45 (20.1)	23 (10.3)	22 (9.8)	
Sep–Nov	156 (31.3)	96 (19.2)	60 (12.0)		73 (32.6)	36 (16.1)	37 (16.5)	
Dec–Feb	113 (22.6)	81 (16.2)	32 (6.4)		46 (20.5)	25 (11.2)	21 (9.4)	
Anterior circulating aneurysm	477 (95.6)	301 (60.3)	176 (35.3)	0.960^a^	216 (96.4)	110 (49.1)	106 (47.3)	0.280^a^
Multiple aneurysms	105 (21.0)	56 (11.2)	49 (9.8)	0.019^a^	45 (20.1)	21 (9.4)	24 (10.7)	0.617^a^
Vitamin D (ng/mL)	17.0 [12.1–22.4]	17.6 [12.6–22.6]	16.3 [11.6–22.3]	0.418^c^	16.0 [11.5–21.3]	15.8 [11.7–21.4]	16.2 [10.8–21.2]	0.904^c^
Vitamin D levels				0.339^a^				0.947^a^
Normal	46 (9.2)	26 (5.2)	20 (4.0)		22 (9.8)	11 (4.9)	11 (4.9)	
Insufficient	133 (26.7)	90 (18.0)	43 (8.6)		48 (21.4)	23 (10.3)	25 (11.2)	
Deficient	320 (64.1)	199 (39.9)	121 (24.2)		154 (68.8)	78 (34.8)	76 (33.9)	

*Note:* Variables are presented as number of patients (%) or median (interquartile range), as appropriate.

^a^ *p*‐values calculated using Pearson's chi‐square test.

^b^ *p*‐values calculated using Fisher's exact test.

^c^ *p*‐values calculated using Mann–Whitney *U* test.

In this study, diabetes was inversely associated with the risk of aSAH (*p* = 0.034; odds ratio [OR], 0.53; 95% confidence interval [CI], 0.290–0.954), whereas multiple aneurysms increased the risk of aSAH (*p* = 0.006; OR, 1.88; 95% CI, 1.197–2.940) in multivariate analysis (Table S1). The prevalence of diabetes was 16.8% (53/315) in patients with unruptured IAs, compared with 9.2% (17/184) in patients with ruptured IAs. Conversely, multiple aneurysms were more prevalent in ruptured IA patients (26.6%, 49/184) than in unruptured IA patients (17.8%, 56/315). Age, sex, BMI, smoking, alcohol use, hypertension, hyperlipidemia, and aneurysm location (anterior circulation) showed no significant association with aSAH in multivariate analysis (*p* > 0.05).

Blood test dates, as presented in Table 1, did not differ significantly between patients with unruptured IA and those with ruptured IA (*p* = 0.147). The median VitD level was 17.0 ng/mL [12.1–22.4]. VitD levels were normal in 9.2% of patients, insufficient in 26.7%, and deficient in 64.1% of patients. No significant difference in VitD levels was observed between patients with unruptured IA and those with ruptured IA (*p* = 0.418). Furthermore, VitD deficiency showed no association with aSAH in this study (*p* = 0.339). To control for confounders, PSM was performed in a 1:1 ratio based on sex, age, BMI, diabetes, hypertension, hyperlipidemia, smoking, alcohol consumption, blood test date, and presence of multiple aneurysms. A total of 112 patients with ruptured IA were successfully matched to 112 patients with unruptured IA. Following PSM, no significant differences were observed in the distribution of the aforementioned variables. VitD levels and deficiency remained unassociated with aSAH (*p* > 0.05).

**FIGURE 2:**
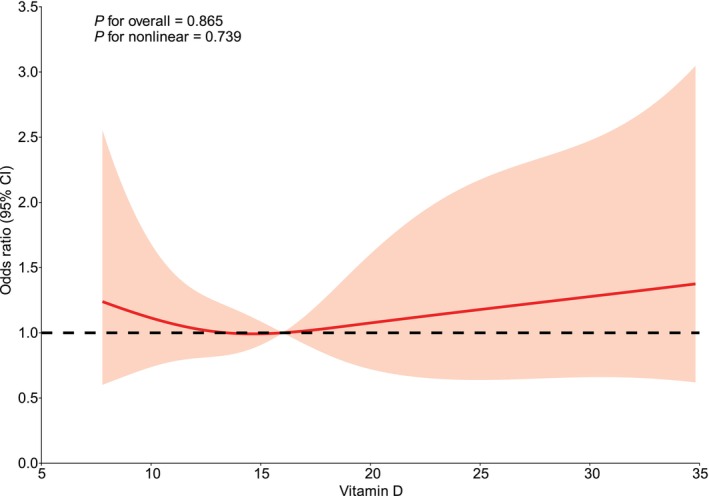
Restricted cubic spline regression model depicting the association between serum vitamin D (VitD) levels and the odds of ruptured intracranial aneurysms.

Additionally, RCS regression analysis showed no significant nonlinear association between serum VitD levels and the risk of IA rupture (*p* = 0.739), nor a significant overall association (*p* = 0.865), further supporting the findings before and after PSM (Figure 2).

### 3.2 The Causal Associations Between Vitamin D Status and IA Rupture in MR Analysis

**FIGURE 3:**
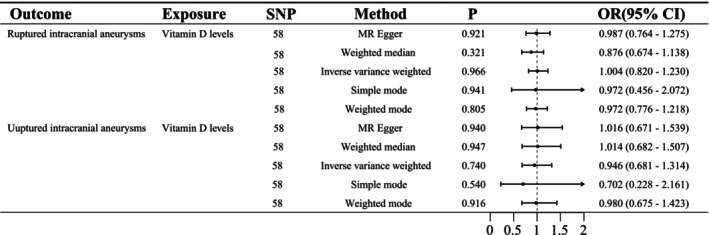
Forest plots for the causal associations of vitamin D (VitD) levels with ruptured intracranial aneurysms or unruptured intracranial aneurysms (UIAs) in discovery cohort.

The IVW‐MR analysis revealed no causal relationship between VitD levels and the risks of aSAH (*p* = 0.966; OR, 1.00; 95% CI, 0.82–1.23) or unruptured IA (*p* = 0.74; OR, 0.95; 95% CI, 0.68–1.31) (Figure 3). These findings remained consistent across multiple MR methods, including weighted median, MR‐Egger, weighted mode, and simple mode approaches, and were replicated in an independent cohort (Table 2). Scatter plots and forest plots further confirmed these findings (Figures S1 and S2). All SNPs used as instrumental variables (IVs) exhibited *F*‐statistics exceeding 20, indicating minimal weak instrument bias (Files S2–, S5). Sensitivity and heterogeneity analyses revealed no evidence of pleiotropy or heterogeneity (Table 3), supporting the robustness and reliability of the MR findings. Leave‐one‐out analysis further validated these results (Figure S3).

**TABLE 2:** The MR results of the replication cohort.

	Exposure	MR method	SNP	*p*	OR (95% CI)
RIA	Vitamin D	MR Egger	93	0.957	0.993 (0.777–1.270)
		Weighted median	93	0.417	0.891 (0.675–1.177)
		Inverse variance weighted	93	0.389	1.076 (0.911–1.270)
		Simple mode	93	0.745	0.907 (0.504–1.633)
		Weighted mode	93	0.948	1.007 (0.821–1.234)
UIA	Vitamin D	MR Egger	93	0.410	0.874 (0.636–1.202)
		Weighted median	93	0.231	0.803 (0.561–1.150)
		Inverse variance weighted	93	0.371	1.103 (0.889–1.369)
		Simple mode	93	0.005	2.910 (1.413–5.990)
		Weighted mode	93	0.596	0.928 (0.706–1.221)

Abbreviations: RIA, Ruptured intracranial aneurysms; UIA, Unruptured intracranial aneurysms.

**TABLE 3:** Heterogeneity and pleiotropy tests of MR in discovery and replication cohort.

Cohort	Test	Outcome	Exposure	Method	Effect size	*p*
Discovery	Heterogeneity	RIA	Vitamin D	Cochran's *Q* test	66.0 (QMR Egger)	0.169
					66.1 (QIVW)	0.192
		UIA	Vitamin D	Cochran's *Q* test	69.8 (QMR Egger)	0.101
					70.2 (QIVW)	0.112
	Pleiotropy	RIA	Vitamin D	MR‐Egger regression	0.001 (Egger regression intercept)	0.825
		UIA	Vitamin D	MR‐Egger regression	−0.005 (Egger regression intercept)	0.580
Replication	Heterogeneity	RIA	Vitamin D	Cochran's *Q* test	110.8 (Q MR Egger)	0.078
					111.7 (Q IVW)	0.080
		UIA	Vitamin D	Cochran's *Q* test	88.6 (Q MR Egger)	0.551
					92.4 (Q IVW)	0.469
	Pleiotropy	RIA	Vitamin D	MR‐Egger regression	0.003 (Egger regression intercept)	0.393
		UIA	Vitamin D	MR‐Egger regression	0.009 (Egger regression intercept)	0.055

Abbreviations: RIA, ruptured intracranial aneurysms; UIA, unruptured intracranial aneurysms.

## 4 Discussion

The relationship between VitD status and the risk of aSAH has received increasing attention due to accumulating evidence demonstrating the high prevalence of VitD deficiency in the population and the potential protective role of VitD in cardiovascular and cerebrovascular health [7, 8, 9, 16]. However, our study, which employed a large case–control design with PSM and two‐sample MR analysis, did not identify any association or causal relationship between VitD status and the risk of IA rupture.

VitD has a potential protective role in maintaining arterial health [17]. VitD receptors are present in vascular smooth muscle and endothelial cells. VitD deficiency is common in patients with IAs, even in those with aSAH and high sun exposure [18]. Although VitD might protect against IAs and reduce the risk of aSAH, our findings did not support this hypothesis, as VitD levels were not inversely associated with aSAH. A previous cross‐sectional study found higher VitD levels in patients with unruptured IAs than in those with ruptured IAs, with VitD independently associated with rupture risk [12]. However, that study had a small sample size and lacked PSM analysis. Our use of PSM balanced baseline characteristics between ruptured and unruptured IA groups, minimizing confounders such as age, sex, and comorbidities. Despite this, we found no significant difference in VitD levels between patients with ruptured or unruptured IA, and VitD deficiency was not associated with aSAH. Our results were consistent with a previous study [10], which found no difference in VitD levels between ruptured and unruptured IA patients, despite higher rates of hypovitaminosis D in IA patients requiring treatment compared to patients without IA. However, our observational study may still be affected by uncontrolled confounders, such as lifestyle, dietary habits, sunlight exposure, and diseases or medications influencing VitD metabolism, which could potentially impact serum VitD levels and our findings. Future studies incorporating detailed lifestyle and environmental data are warranted to better account for these influences.

To address this limitation, we performed MR analysis, which utilizes genetic variants as instrumental variables to infer causal relationships. This analysis revealed no causal association between genetically determined VitD levels and IA rupture, consistent with prior MR studies utilizing distinct GWAS cohorts that also found no causal effect of VitD on IA rupture. We further used a restricted cubic spline regression model to evaluate potential nonlinear associations between serum VitD levels and IA rupture risk. The analysis revealed no significant linear or nonlinear relationship, thereby reinforcing our findings. These results suggest that previously reported associations may be attributable to residual confounding or unmeasured biases. Although lower VitD levels were frequently observed in aneurysm patients, a recent meta‐analysis of VitD intervention trials failed to demonstrate a significant reduction in cardiovascular events with VitD supplementation, suggesting that lower VitD levels may represent an epiphenomenon rather than a causative factor [19]. One recent study reported VitD deficiency could promote IA rupture in a murine model [20]. However, all SAH murine models have inherent limitations, and the specific animal model used in that study may not fully replicate human aneurysm rupture mechanisms. Therefore, these findings cannot be directly extrapolated to humans.

The biological mechanisms through which VitD influences vascular health may not directly relate to the pathophysiology of IA rupture. Although VitD may contribute to general vascular maintenance, the specific processes leading to aneurysm formation and rupture—such as hemodynamic stress, vascular wall degeneration, and inflammatory infiltration—may be less affected by VitD levels. Additionally, the VitD threshold for vascular protection may be lower than deficiency levels, meaning even “low” VitD levels could suffice for vascular integrity. However, studies indicate that VitD has anti‐inflammatory effects [21], and cerebrovascular inflammation is a major pathological factor for aneurysm rupture. Low VitD levels are also associated with risk factors for aSAH, such as hypertension, hyperlipidemia, and hyperhomocysteinemia [22]. Therefore, our findings should be interpreted with caution. An alternative explanation is that the relationship between VitD and IA rupture is more complex than a linear association. VitD may directly or indirectly influence measurable and unmeasurable factors that contribute to the pathological progression of aneurysms. Its vascular effects might also be more pronounced in specific subgroups, such as individuals with certain comorbidities or genetic predispositions, and these effects may have been attenuated in our overall study population.

Although our study did not identify a significant association between VitD levels and IA rupture, this finding does not rule out VitD's potential role in other aspects of aneurysm biology, such as formation or progression. Future studies should investigate these areas through longitudinal study designs or by focusing on subgroups more susceptible to the effects of VitD. Further research should also explore interactions between VitD and other factors, such as genetic variation, environmental exposures, or comorbidities, which may modulate IA rupture risk. For instance, VitD might exert protective effects only in individuals with specific genetic profiles or under particular environmental conditions. Elucidating these interactions could provide deeper insights into VitD's role in vascular pathophysiology.

The strengths of our study include the utilization of PSM and MR analysis to mitigate confounding and establish causal inferences. PSM effectively balances control groups and reduces selection bias in observational studies, while MR reliably evaluates causality by employing genetic variants as instrumental variables for VitD levels, thereby minimizing confounding and reverse causation. However, the MR analysis design differs from our observational analysis. While the observational study compared VitD levels in patients with ruptured and unruptured IA, the MR analysis assessed genetically predicted VitD levels in relation to unruptured IA and aSAH risk versus general population controls. Nevertheless, these complementary methods provide both real‐world and genetically informed insights into the potential role of VitD in the pathophysiology of IA.

However, our study has several limitations. First, while PSM balanced observed baseline characteristics, unmeasured confounders may have introduced bias. Second, MR analysis assumes the genetic variants used as IVs are associated with VitD levels but not with other factors affecting rupture risk. Third, although we selected genetic variants strongly associated with VitD levels, they may have unexplained pleiotropic effects. Fourth, MR assumes a linear relationship between VitD levels and rupture, which may not hold if a threshold or nonlinear effect exists. Additionally, other clinical factors, including age, diabetes, and multiple aneurysms, were significantly associated with aSAH in our observational study but were excluded from MR analysis due to limited genetic instruments and research focus. Moreover, our MR analysis utilized GWAS data from European populations, whereas our observational cohort was Chinese. Genetic differences in VitD metabolism, such as SLC24A5, CYP2R1, and GC variants between Europeans and Asians, may limit the generalizability of our MR findings [23, 24]. Dietary, lifestyle, and environmental factors, such as sunlight exposure and pollution, could also affect VitD‐related genotype expression. Future studies using East Asian GWAS data are needed to validate these results in more genetically similar cohorts. Finally, not all relevant SNPs were included, which may have influenced the robustness of causal inference. A large‐scale prospective study with long‐term follow‐up is still needed to confirm our findings.

## 5 Conclusion

Our study did not demonstrate an association or a causal relationship between VitD levels and aneurysmal subarachnoid hemorrhage (aSAH). The inverse associations observed in previous cohort studies may be attributable to confounding factors or reverse causation. Although VitD deficiency is common in patients with aSAH, supplementation may not prevent aneurysm rupture.

## Ethics Statement

The study was approved by the ethics committee of Qilu Hospital of Shandong University, and written informed consent was obtained from all the patients before enrollment.

## Conflicts of Interest

The authors declare no conflicts of interest.

## Supporting information


**Figure S1:** Scatter plots of the relationship between vitamin D levels and ruptured or unruptured intracranial aneurysms. Scatter plots illustrate the associations between vitamin D levels and ruptured or unruptured intracranial aneurysms in the discovery cohort (A and C) and the replication cohort (B and D). MR, Mendelian randomization; SNP, single nucleotide polymorphism.
**Figure S2:** Causal effects of individual SNPs in MR analyses. Forest plots show the estimated MR effect sizes of individual SNPs on ruptured or unruptured intracranial aneurysms by vitamin D levels in the discovery cohort (A and C) and the replication cohort (B and D). MR, Mendelian randomization; SNP, single nucleotide polymorphism.
**Figure S3:** Leave‐one‐out sensitivity analysis of MR results. Leave‐one‐out sensitivity analyses are shown for the discovery cohort (A and C) and the replication cohort (B and D). MR, Mendelian randomization.

**File S1:** cns70617‐sup‐0002‐FileS1.xlsx.

**File S2:** cns70617‐sup‐0003‐FileS2.xlsx.

**File S3:** cns70617‐sup‐0004‐FileS3.xlsx.

**File S4:** cns70617‐sup‐0005‐FileS4.xlsx.

**File S5:** cns70617‐sup‐0006‐FileS5.xlsx.

**Table S1:** Binary logistic regression between patients with ruptured intracranial aneurysms and patients with unruptured intracranial aneurysms.

## Data Availability

The raw data supporting the conclusions of this article will be made available by the authors, without undue reservation.
