# Supplementary material for: Serum Vitamin D Levels Were Not Associated With the Risk of Aneurysmal Subarachnoid Hemorrhage: A Large Cohort Study With Propensity Score Matching and Mendelian Randomization Analysis
Source: CNS Neurosci Ther. 2025 Sep 30;31(10):e70617. doi: 10.1111/cns.70617 (PMC12481830; doi:10.1111/cns.70617)
Supplement: Supplementary file 1 — Figure S1: Scatter plots of the relationship between vitamin D levels and ruptured or unruptured intracranial aneurysms. Scatter plots illustrate the associations between vitamin D levels and ruptured or unruptured intracranial aneurysms in the discovery cohort (A and C) and the replication cohort (B and D). MR, Mendelian randomization; SNP, single nucleotide polymorphism. Figure S2: Causal effects of individual SNPs in MR analyses. Forest plots show the estimated MR effect sizes of individual SNPs on ruptured or unruptured intracranial aneurysms by vitamin D levels in the discovery cohort (A and C) and the replication cohort (B and D). MR, Mendelian randomization; SNP, single nucleotide polymorphism. Figure S3: Leave‐one‐out sensitivity analysis of MR results. Leave‐one‐out sensitivity analyses are shown for the discovery cohort (A and C) and the replication cohort (B and D). MR, Mendelian randomization. [file CNS-31-e70617-s004.docx]

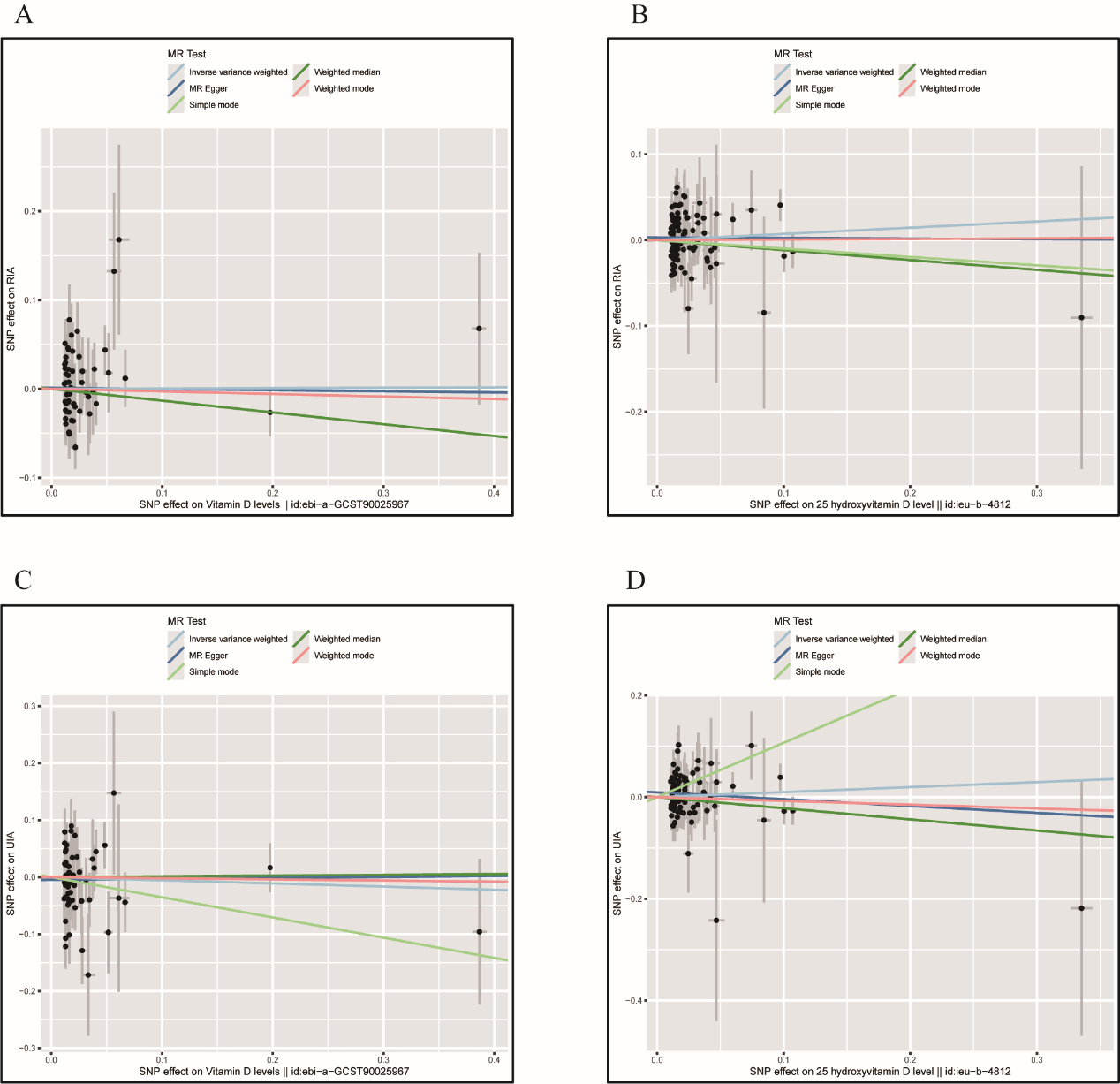


**Supplementary Figure 1.** Scatter plots of the relationship between vitamin D levels and ruptured or unruptured intracranial aneurysms. Scatter plots illustrate the associations between vitamin D levels and ruptured or unruptured intracranial aneurysms in the discovery cohort (A and C) and the replication cohort (B and D). MR, Mendelian randomization; SNP, single nucleotide polymorphism.


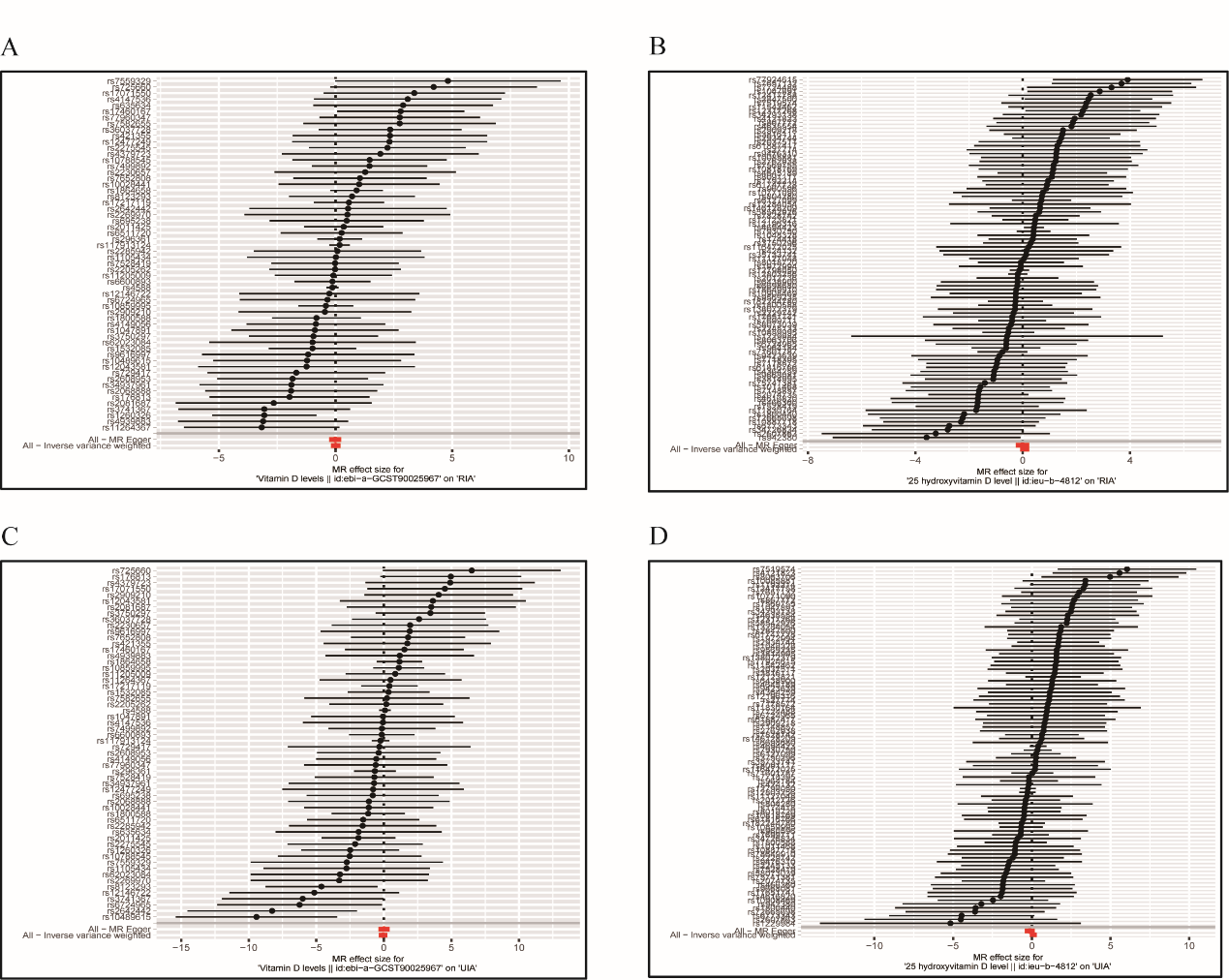


**Supplementary Figure 2.** Causal effects of individual SNPs in MR analyses. Forest plots show the estimated MR effect sizes of individual SNPs on ruptured or unruptured intracranial aneurysms by vitamin D levels in the discovery cohort (A and C) and the replication cohort (B and D). MR, Mendelian randomization; SNP, single nucleotide polymorphism.


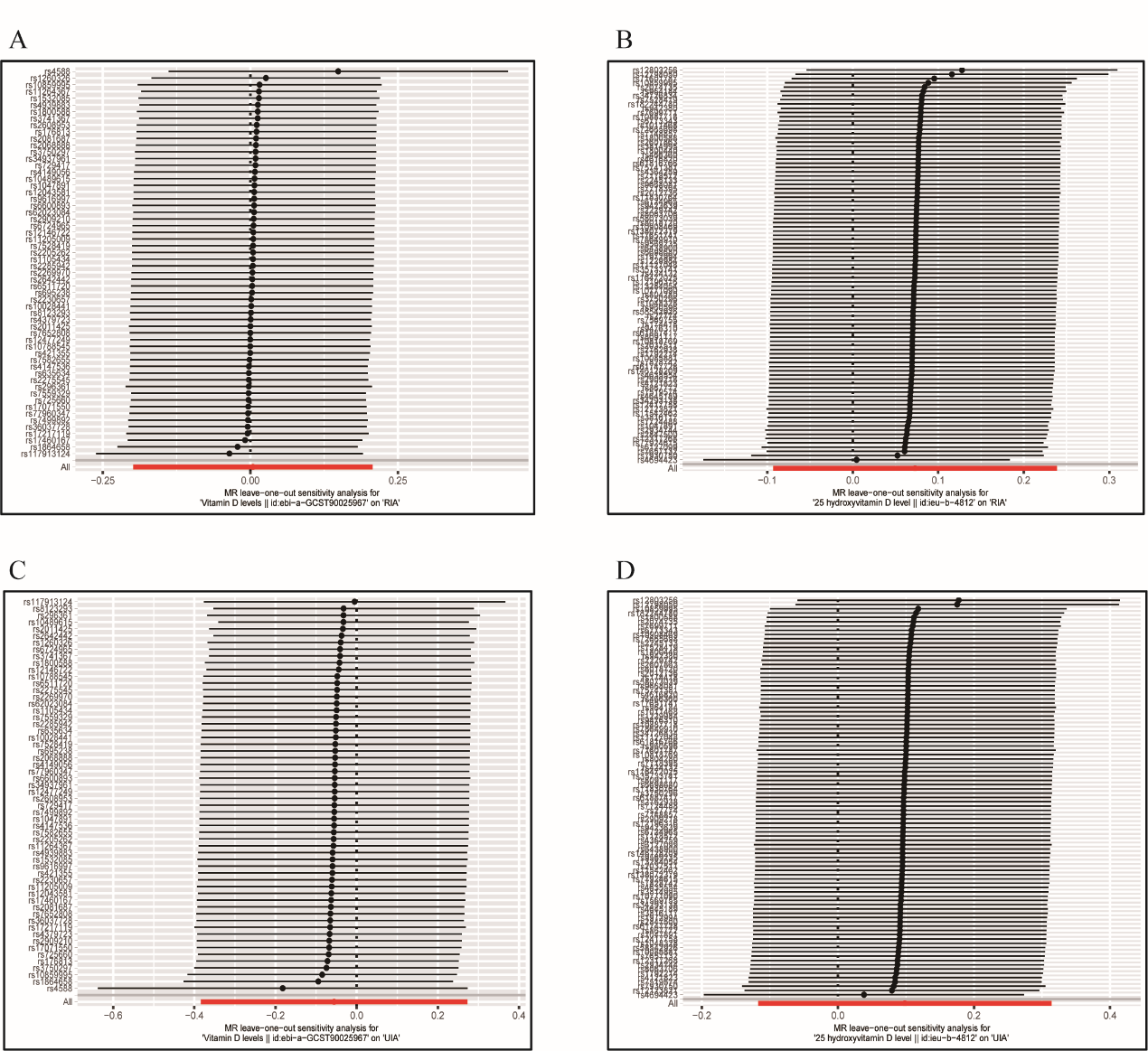


**Supplementary Figure 3.** Leave-one-out sensitivity analysis of MR results. Leave-one-out sensitivity analyses are shown for the discovery cohort (A and C) and the replication cohort (B and D). MR, Mendelian randomization.
