# Supplementary material for: Serum Vitamin D Levels Were Not Associated With the Risk of Aneurysmal Subarachnoid Hemorrhage: A Large Cohort Study With Propensity Score Matching and Mendelian Randomization Analysis
Source: CNS Neurosci Ther. 2025 Sep 30;31(10):e70617. doi: 10.1111/cns.70617 (PMC12481830; doi:10.1111/cns.70617)
Supplement: Supplementary file 7 — Table S1: Binary logistic regression between patients with ruptured intracranial aneurysms and patients with unruptured intracranial aneurysms. [file CNS-31-e70617-s002.docx]

Supplemental Table. Binary logistic regression between patients with ruptured intracranial aneurysms and patients with unruptured intracranial aneurysms.

| **Variable** | ***p*** | **OR(95%CI)** |
| --- | --- | --- |
| Age≤50 | Reference |  |
| Age51-60 | 0.108 | 1.86 (0.872-3.969) |
| Age61-70 | 0.531 | 1.26 (0.611-2.600) |
| Age>70 | 0.662 | 0.85 (0.408-1.767) |
| Diabetes | 0.034 | 0.53 (0.290–0.954) |
| Multiple aneurysms | 0.006 | 1.88 (1.197–2.940) |
